# A Review on the Video-Based River Discharge Measurement Technique

**DOI:** 10.3390/s24144655

**Published:** 2024-07-18

**Authors:** Meng Chen, Hua Chen, Zeheng Wu, Yu Huang, Nie Zhou, Chong-Yu Xu

**Affiliations:** 1 State Key Laboratory of Water Resources Engineering and Management, Wuhan University, Wuhan 430072, China; mechen@whu.edu.cn (M.C.); wuzeheng@whu.edu.cn (Z.W.); sifmol@whu.edu.cn (Y.H.); niezhou@whu.edu.cn (N.Z.); 2 Department of Geosciences, University of Oslo, N-0316 Oslo, Norway; c.y.xu@geo.uio.no

**Keywords:** video, river discharge measurement, flow monitoring, image recognition

## Abstract

The hydrological monitoring of flow data is important for flood prevention and modern river management. However, traditional contact methods are increasingly struggling to meet the requirements of simplicity, accuracy, and continuity. The video-based river discharge measurement is a technique to monitor flow velocity without contacting the water body by using the image-recognition algorithms, which has been verified to have the advantages of full coverage and full automation compared with the traditional contact technique. In order to provide a timely summary of the available results and to inform further research and applications, this paper reviews and synthesizes the literature on the general implementation routes of the video-based river discharge measurement technique and the principles and advances of today’s popular image-recognition algorithms for velocity detection. Then, it discusses the challenges of image-recognition algorithms in terms of image acquisition conditions, parameter uncertainties, and complex meteorological and water environments. It is concluded that the performance of this technique can be improved by enhancing the robustness and accuracy of video-based discharge measurement algorithms, minimizing weather effects, and improving computational efficiency. Finally, future development directions for further perfecting this technique are outlined.

## 1. Introduction

River discharge is one of the fundamental hydrological elements, and monitoring flow data is of paramount importance for both flood disaster prevention and water resource management. Due to the harsh field environment and the complex hydraulic characteristics of rivers, flow measurements have always been difficult to implement. The conventional discharge measurement technique relies on in situ velocity measurements using current meters, floats, or Acoustic Doppler Current Profiler (ADCP) for discharge estimation. However, these contact-based approaches have been proven to be inadequate when confronting the growing occurrence of severe floods, demanding excessive time and labor resources [1].

For safety reasons, it is not easy to deploy current meters in water during high flood scenarios. Moreover, these measurements have the potential to disrupt the natural flow of the river and damage the instruments when exposing them to sediment and floating debris [2]. Employing floats for flow measurement is a straightforward yet precarious method. Floats are susceptible to entrapment by locally generated eddies, resulting in potential measurement inaccuracies [3]. ADCP has emerged as the predominant approach for flow measurements. However, measurements are subject to large errors in the presence of high turbulence, aeration, and bed movement. Additionally, the high cost of equipment acquisition and management has prevented many stations from adopting it [4].

In pursuit of more timely, secure, and intelligent flow data acquisition, non-contact flow measurement techniques utilizing acoustics, radar, and remote sensing have been developed in recent decades, significantly diversifying the field of flow assessment [5,6,7,8,9,10,11,12,13,14,15]. Technology based on the acoustic time difference is commonly used in measuring industrial polluted water flow. van Willigen et al. [5] made an algorithmic improvement for the zero flow error problem of a time-difference ultrasonic flowmeter. Considering the effect of water scouring slopes on transducers, the interference of underwater vegetation on sound wave propagation, etc., the technology has fewer applications in field river measurements [16]. Electromagnetic wave flow measurements are widely used in the emergency monitoring of rivers, which is divided into two forms, namely obtaining the point velocity of the electric current meter flow measurement and obtaining the flow velocity field of the side-scanning radar flow measurement. However, the accuracy is not good in the low-flow conditions, and the cost of multiple instruments needs to be considered for point-flow measurements [7]. At last, Satellite-based flow measurement is an emerging cross-cutting application of hydrology and remote sensing [12]. The correlation between the river flow and the remote sensing inundation range has made this technology have great potential [10]. However, the experimental accuracy of current studies in this area falls short of that required for hydrological measurements. Besides, the method relies too heavily on ground measurement information and historical data [10,11].

In recent years, rapid advancements in computer vision and artificial intelligence technologies, along with the widespread utilization of intelligent video surveillance tools, have propelled the development of image recognition technology [17,18]. This has brought a fresh perspective for hydrological monitoring, and hydrological monitoring based on image recognition technology has gradually become a vital research direction. The idea behind using video images is to assume that surface flow features consisting of surface ripples or floating objects would follow the surface velocity when the wind effect is negligible [19]. Video image processing research focuses on the removal of noise and the correction of images to extract a clear texture pattern reflecting the vector trajectory of the flow field during the time period, whereby real-time fast analysis of data streams can be realized.

Research is currently focusing on the study and improvement of image-recognition algorithms, and much work has been carried out by several teams in different countries [19,20,21,22,23,24,25,26,27,28]. Fujita et al. [20] first improved the particle image velocimetry (PIV) technique of indoor experiments and applied it to the observation of the surface flow field and flow measurement of rivers in the field. Then, this technique, which was named large-scale particle image velocimetry (LSPIV), attracted wide attention [21,22,23]. After more than a decade, Muste et al. [24] provided a comprehensive review of LSPIV research progress and discussed examples of technical configurations, including space–time image velocimetry (STIV), large-scale adaptive PIV (LSAPIV), real-time LSPIV (RTLSPIV), LSPIV simulator, mobile LSPIV, controlled surface wave image velocity (CSWIV), and river digital mapping (RDM), each of which was developed to address a specific purpose. To overcome the dependence of LSPIV on tracer particles and to improve the computational efficiency, Fujita et al. [3] proposed STIV utilizing natural ripple tracers. Then, this team conducted in-depth research on the problems of illumination limitations, river width limitations, and image noise interference suffered by STIV [19,25,26], gradually improving this velocity measurement method and laying the foundation for the subsequent proposal and improvement of the video-based discharge calculation algorithm.

**Figure 1:**
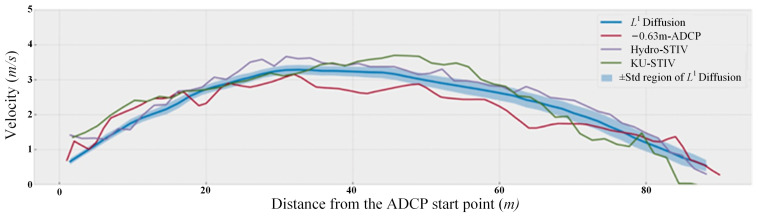
Comparison of velocity distribution inferred from different algorithms along the channel section. The Hydro-STIV [29] and the KU-STIV [3] represent the surface velocity acquired from different software based on the STIV method, and the L^1^ Diffusion [1] is the velocity acquired from variational method-used video [1].

The method of measuring free surface velocity through river imagery presents an innovative approach to flow measurements. An analysis of video images acquired from the river surface enables the safe and convenient determination of surface velocity distribution, facilitating the estimation of cross-sectional flow. It is reliable in terms of accuracy, and a comparison with the ADCP is shown in Figure 1. In addition, it has superior performance compared with the traditional discharge measurement technique in terms of monitoring efficiency, convenience, and environmental applicability [1,24]. It is the best alternative for scenarios such as extreme flood conditions where an observation is inconvenient, measuring reaches without hydrological stations, and measuring small rivers in mountainous areas (high flow variability, shallow water, and rocky riverbed). Furthermore, the installation of river video capture equipment enables the real-time monitoring of flow conditions and storage of large amounts of two-dimensional information, which allows for easy access to historical and current data. Presently, the imperative need arises for the advancement of a novel generation of non-contact, real-time, and efficient flow monitoring instruments. Video-based discharge measurements promise to elevate existing hydrological survey capabilities and fulfill the requirements of cost-effectiveness, precision, and adaptability in contemporary flow measurements.

The past decade’s scientific literature details the development and application of the video-based discharge measurement technique, accumulating substantial expertise in diverse methods. A review of these methods is timely and needed to summarize what we have learned from past studies, as well as conclusions that can guide the future endeavors both in research and practical applications. Therefore, this paper aims to provide a comprehensive review of the recent progress, summarize contemporary research findings, and encourage further exploration and applications in video-based river discharge measurements and the related domains. The rest of the paper is organized as follows. Section 2 provides an overview of video-based discharge measurements with a brief description of the process concerning river surface velocity measurements and discharge calculations. Section 3 details the current state of the development of surface velocity identification algorithms and the four classes of algorithms. Based on this, Section 4 assesses the challenges of video-based river discharge measurements, including the key issues and application potential of the technique. Finally, Section 5 summarizes the research progress of video-based discharge measurements and looks forward to the development prospect of video-based discharge measurement technology.

## 2. Outline and Methodological Process

The core of the river discharge measurement process is the acquisition of velocities at the test section, which can be used in conjunction with section information to estimate instantaneous discharge. As a non-contact method, the video-based method uses a video camera as a sensor to directly perceive the surface of the water body. Luminance variations stemming from water surface floating objects, man-made particles, or naturally occurring ripples, among other flow features, are regarded as water tracers. Assuming that they follow the same motion pattern as the river water body when the breezy influence is ignored, the velocity of the water body can be explicitly visualized in this way.

**Figure 2:**
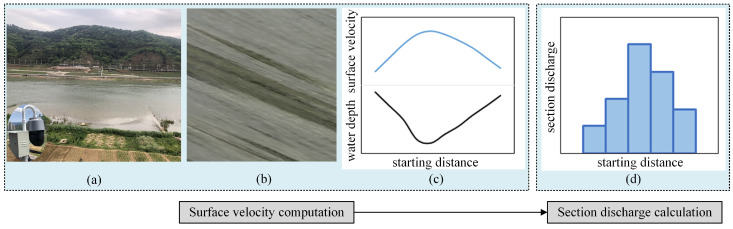
A typical workflow of a video-based river discharge measurement technique: (**a**) capturing water surface images using cameras; (**b**) extracting the tracer information (e.g., STIV); (**c**) calculating the velocity distribution; and (**d**) summing the subsection discharges.

The basic idea of the visualization-based discharge calculation can be divided into two steps (Figure 2): the first step is the process of river surface velocity computation, which involves capturing water surface images (Figure 2a), recognizing the tracer information and extracting them from the original photographs (Figure 2b), and then calculating the velocity distribution (Figure 2c). The second step pertains to the discharge calculation mechanism. It involves the estimation of the mean velocity distribution along the bathymetry based on surface velocity and the determination of the discharge magnitude by referencing the pre-test acquisition of the channel cross-section’s shape and dimensions (Figure 2d). It should be noted that the accuracy of the results needs to be validated by synchronizing comparative test trials using a basic current meter or ADCP.

### 2.1. Basic Process for Calculation of River Surface Velocity

Suitable water surface images are essential to ensure calculation accuracy. The usual choice for collecting flow images is to set up fixed cameras along the surveyed riverbanks and to arrange for proper communication and power supply lines. The camera needs to define the resolution, frame rate, and other parameters. The commonly used sampling frequency is 24–60 Hz, which should be adjusted according to the river flow velocity. Pizarro et al. [30] proposed two criteria for the optimal number of frames and concluded that selecting fewer consecutive shots can reduce the image velocimetry error. However, a challenge arises when using a fixed camera to capture the distant flow field due to low spatial resolution. To address this issue, Kim et al. [31] designed a vehicle-mounted camera system that offered flexibility by allowing for the adjustment of the camera’s angle through the control of the head’s rotation on top of the vehicle. Additionally, Bechle et al. [32] developed a measurement system based on binocular imaging, using two cameras to capture the near and far fields of a river and synchronizing the sampling via a signal generator. With the development of aerial observation technology, media helicopters and unmanned aerial vehicles (UAVs) began to be used for image acquisition. The cameras are able to take continuous aerial photographs, which are very suitable for short-term observation situations, and the range and stability of image acquisition have been greatly improved [33,34,35,36,37]. Based on UAV technology, Biggs et al. [38] developed a novel aerial tracer particle distribution system that provided a potential solution to the problem of identifying flow velocities in cases of low-flow or a lack of tracers in man-made channels.

**Figure 3:**
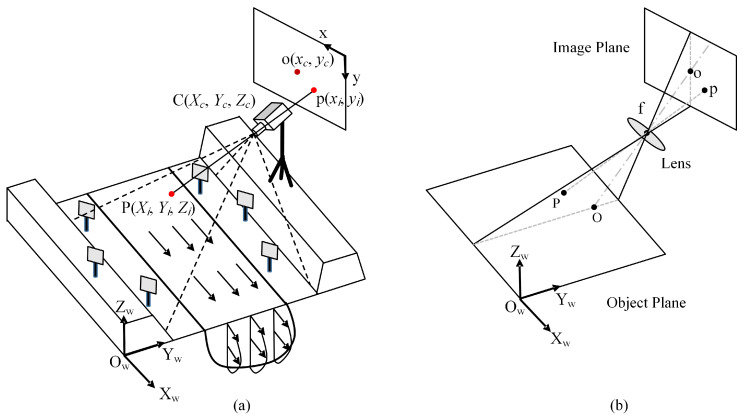
Model of center perspective projection under an oblique view: (**a**) on-site measurements situation using a camera; and (**b**) geometric model.

Water surface images captured directly by the camera often require processing to mitigate the effects of lens distortion, shadows, and flares. This processing typically involves ortho-rectification and filtering to eliminate background noise and enhance particle tracing. The theoretical basis for ortho-rectification is the center perspective projection model, and Figure 3 illustrates the imaging mechanism when taking an image of a river. The dashed line in Figure 3a defines the camera’s imaging area, where o is the image center point and p is a point within the area.

Ortho-rectification is a resampling process and relies on the ground control points (GCPs). The parallax caused by the ground elevation is eliminated by calibrating the conversion parameters to link the world coordinates (*W*-*XYZ*) with the image plane (*I*-*xy*) to enhance the analysis of the velocity field [39]. The transformation relationship given by Equation (1) can be obtained from the covariance equation of the center perspective projection model:

(1) $$\left[\begin{array}{l} x\\ y \end{array}\right]=\left[\begin{array}{cccc} C_{11} & C_{12} & C_{13} & C_{14}\\ C_{21} & C_{22} & C_{23} & C_{24} \end{array}\right]\left[\begin{array}{c} X\\ Y\\ Z\\ 1 \end{array}\right]$$

where *x*, *y* represent the image pixel coordinates, *X*, *Y*, *Z* correspond to the world physical coordinates, and *C*_11_~*C*_24_ are parameters in the coordinate system transformation matrix to achieve the conversion between two coordinates.

The form used to compute and solve the transformation matrix is usually the Direct Linear Transformation (DLT) as Equation (2):

(2) $$\left\{\begin{array}{l} x=x_{c}+f\frac{r_{11}(X-X_{c})+r_{12}(Y-Y_{c})+r_{13}(Z-Z_{c})}{r_{31}(X-X_{c})+r_{32}(Y-Y_{c})+r_{33}(Z-Z_{c})}\\ y=y_{c}+f\frac{r_{21}(X-X_{c})+r_{22}(Y-Y_{c})+r_{23}(Z-Z_{c})}{r_{31}(X-X_{c})+r_{32}(Y-Y_{c})+r_{33}(Z-Z_{c})} \end{array}\right.$$

where (*x_c_*, *y_c_*) denotes the phase plane coordinates of the image center point, (*X_c_*, *Y_c_*, *Z_c_*) represents the actual spatial right-angle coordinates of the camera, *f* is the focal length of the camera, and *r_ij_* (*i*, *j* = 1~3) is the conversion coefficient between the image coordinate system and the world coordinate system.

The above formula contains 12 unknown transformation parameters, necessitating a minimum of 6 sets of GCPs (*x*, *y*) and (*X*, *Y*, *Z*). In practice, 6 or more calibration plates are set up at the shooting site (e.g., the grey plates in Figure 3). Then, with a total station to measure their world coordinates relative to the measurement site and using the video image to obtain the image coordinates, these coordinates will be substituted into the formula so the transformation coefficients can be obtained. Upon establishing the transformation relationship and assuming a horizontal water surface, the generation of a non-strain image finally becomes feasible. This facilitates the derivation of world coordinates for velocimetry points, based on the image coordinates of their initiation and termination points. The limitations of this method are not taking into account the optical aberrations of the non-measuring camera and the dynamic changes in the water surface elevation. To address these limitations, Li et al. [40] developed a stereo-imaging LSPIV system (SI-LSPIV), which reconstructs the three-dimensional topography and water surface distribution using a point cloud, leading to improved measurement accuracy.

Another key point in processing water surface images is filtering. Contrasting the simulated laboratory environment, which boasts minimal interference and powerful lasers, the natural river surface is characterized by dimmer illumination and more intricate conditions. This results in excessive information that deteriorates image quality. To counter this, image-enhancement techniques, such as histogram equalization, smoothing, and edge detection can be employed to compress the image size while effectively eliminating noise [41,42,43,44,45].

**Figure 4:**
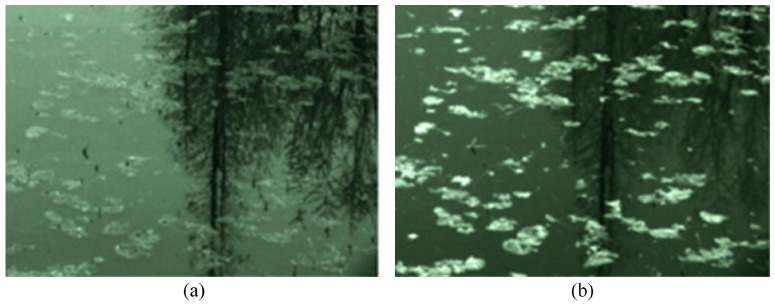
River surface images with different spectral bands [46]: (**a**) full-spectrum band; and (**b**) NIR band.

The commonly used denoising tools are filters and spectra. Zhang et al. [46] developed a near-infrared (NIR) smart camera combining NIR imaging and spatial high-pass filtering (SHPF) methods for image acquisition and preprocessing, which improved the peak signal-to-noise ratio (PSNR) of the estimated motion vectors (Figure 4). SHPF can effectively eliminate background structures and further reduce noise by subtracting offset values, thus increasing the percentage of correct vectors in the instantaneous flow field. Fujita et al. [20] used sharpening filtering and histogram equalization to enhance the contrast of water surface images with a spatial-domain correlation matching method, and introduced the standard filter (STD) and 2D auto-correlation function method to process images [26]. More recently, Zhao et al. [4] utilized the canny operator for the edge detection of images to suppress the background and proposed a new frequency-domain-based filtering technique to eliminate noise and interference in the original image. Lu et al. [47] designed and employed Multi-Scale Retinex (MSR), the fourth-order Gaussian derivative, a noise suppression function, and a directional filtering function to enhance the structure of the image texture, and finally Fourier Maximum Angle Analysis (FMAA) was utilized to further filter out the noise and extract the key information.

As it is not easy to obtain suitable images of water flow, large datasets of river environments are also of interest, which will effectively contribute to the development of image velocimetry methods and further scientific assessments. Perks et al. [48] collated and described datasets obtained from seven countries in Europe and North America, including videos subjected to a series of pre-processing and image velocimetry analyses, for a practical reference.

Once high-quality images of the river have been obtained, various image recognition algorithms can be employed to calculate the surface flow velocity. The simpler one is the coordinate image-processing method, an improvement in the traditional buoy method. In this method, a camera is used to take two consecutive shots of a buoy and record the camera interval. Then, the distance moved by the buoy is calculated from the photos, and thus, the surface velocity can be obtained by dividing the two [49]. There are four main categories of methods that have been studied: computational methods based on particle image recognition [24], space–time images of water flow [3], probability and variational methods [50], and deep learning methods [27]. These algorithms will be elaborated on in Section 3. Some of the methods may also have to go through a stage of flow field post-processing to further correct the erroneous velocity vectors and finally obtain the solved surface flow velocity.

### 2.2. Main Principle of River Discharge Estimation

**Figure 5:**
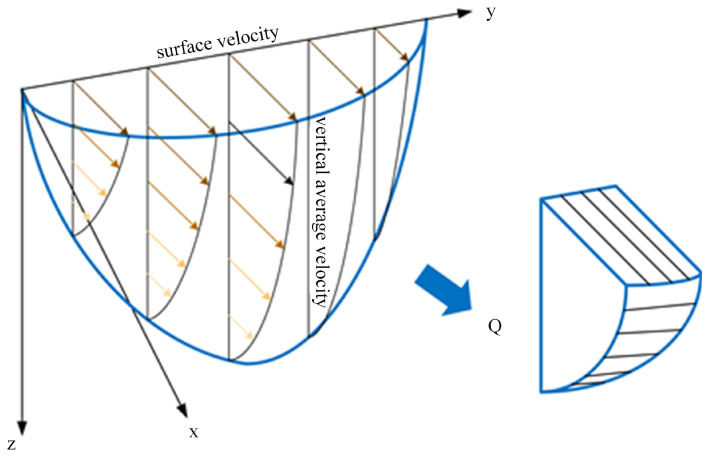
Discharge calculation based on the velocity-area method.

In recent years, methods for computing river cross-section discharge using surface velocity have evolved. Representative is the probabilistic conceptual method, which indirectly estimates discharge by leveraging auxiliary measurements at the gauging station or historical hydrological data [51,52]. However, the most prevalent method remains the velocity-area technique, reliant on surface velocity coefficients [53,54]. The computerized system is based on the measured surface velocity converted to estimate the average velocity of each vertical line and reference to the shape and size of the channel section obtained before the test to measure the size of the sub-section discharge. The cross-sectional discharge is then obtained by summing the discharge of each sub-section, as shown in Figure 5. The discharge calculation formulas are as Equations (3) and (4).

(3) $$v^{\prime }=\alpha \times v$$

(4) $$Q_{ms}=\sum_{i=1}^{n}Q_{i}=\omega A_{1}{v^{\prime }}_{1}+\sum_{i=2}^{n-1}A_{i}\frac{{v^{\prime }}_{i-1}+{v^{\prime }}_{i}}{2}+\omega A_{n}{v^{\prime }}_{n-1}$$

where *v* is the surface velocity, *v*′ is the average velocity of each corresponding vertical line, and the surface velocity coefficient *α* is the ratio of the vertical average velocity to the surface velocity, which is mainly affected by the distribution pattern of the vertical velocity and takes a value usually varying between 0.70 and 0.93 [55]. The vertical line divides the cross-section into *n* parts, the flow enclosed by each part is *Q_i_*, the area is *A_i_*, and *ω* is the bank flow coefficient, ranging from 0 to 1, with steeper slopes approaching 1.

Given the presence of turbulent flow regimes, including stagnant water and backwater areas near the bank boundary, and the reduction of the camera’s view field under high water conditions, potential measurement blind zones may occur near both banks. Therefore, it is prudent to introduce a blind zone discharge coefficient, denoted as *B_z_*, to correct errors in discharge measurement results due to measurement blind zones. The sectional discharge is estimated based on the ratio of the measurement zone’s discharge area to the total discharge area, as shown in Equation (5).

(5) $$Q_{cb}=Q_{ms}+Q_{ms}(A_{cb}/A_{ms}-1)B_{z}$$

where *Q_cb_* is the calibrated complete cross-section discharge, *Q_m__s_* is the measured cross-section discharge in the view field, *A_cb_* is the total discharge area, *A_ms_* is the discharge area of the measurement area, and *B_z_* is the blind discharge coefficient, which is related to the cross-section topography and water level and needs to be calibrated based on measured discharge information obtained using other methods.

## 3. Algorithm Classification and Progress

### 3.1. Algorithms Based on Particle Image Recognition

The model for identifying velocities using particle images is based on the distribution of tracer particles and the correlation of image frames. It centers on the assumption that particles are well-followed and calculates the velocity vectors at each point by matching the neighboring spaced particles and inferring the higher-order motion parameters. Two methods are more successful in early research in the laboratory: particle image velocimetry (PIV) [56] and particle tracking velocimetry (PTV) [57,58]. These methods can leverage the continuous motion image of particles to provide information on the instantaneous flow field. The algorithmic mechanism of video frame combining and feature matching has shown a high potential for use in the automation and real-time measurements [57,59,60]. Based on PIV, Fujita and Komura [61] attempted to extend the method to outdoor rivers and then proposed the large-scale particle image velocimetry (LSPIV) and successfully used it for flow field observations and flow measurement in the Yodo River [20]. LSPIV subsequently received widespread attention due to its excellent performance in monitoring river flow under extreme field conditions [24].

**Figure 6:**
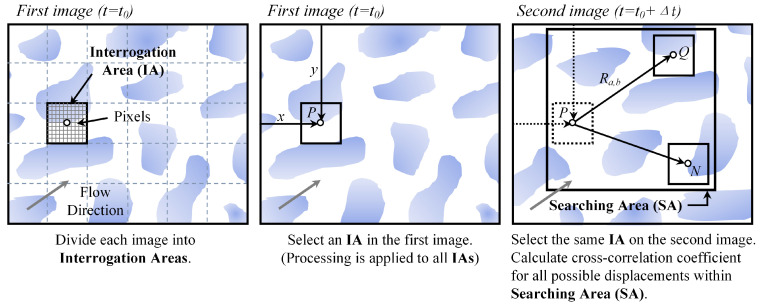
Conceptualization of the LSPIV image-processing algorithm (the patterns in the images above are usually formed by the clustering of smaller particles of the same nature, i.e., foam, leaves, or artificial seeding added to the surface for collecting the measurements) [24].

A common algorithm implemented in LSPIV is mutual correlation: (1) two images with a known time interval of Δt are extracted and processed as input from the streaming video; (2) a block of source pixels called the interrogation area (IA) is read in the previous image; (3) correlation is computed for a window of the same size within a larger search area (SA) selected in the next one, and the pair of windows with the largest value is assumed to be the most probable displacement of the pattern between the two consecutive images (Figure 6); (4) finally, the average displacement of the particles within the window is calculated, and the velocity vector is obtained by dividing the displacement vector by Δt. The size of SA may be as large as the image and can be defined by the user, but it is usually limited by the measurement or estimation of the maximum velocity expected on the water surface [62]. The expression of the 2D discrete cross-correlation function is as Equation (6). Then, numerous studies have been conducted to improve LSPIV, as shown in Table 1.

(6) $$R_{a,b}=\sum_{i=1}^{M_{x}}\sum_{j=1}^{M_{y}}M(i,j)\cdot B(i+a,j+b)$$

where *M* (*i*, *j*) and *B* (*i*, *j*), respectively, denote the intensity of (*i*, *j*) pixels within the window in the before and after images. The value of *R* is computed at each coordinate (*a*, *b*), and due to the limit of the SA size, the larger the value, the closer it is to the real displacement of the particle.

Overall, particle-based image velocimetry is a typical non-contact video discharge measurement technique. The algorithm represented by LSPIV has better flow field estimation in the case of sufficiently large and well-followed particles. However, it comes with the drawback of substantial computational requirements and limited accuracy in the presence of glare and shadows on the river surface. SSIV reduces the influence of the error vectors on its basis, but all such methods almost inevitably encounter the problem of having a finite and uncontrollable supply of natural tracer particles. It is also worth noting that PTV-related methods have the unique advantage that the analyst can control the obtained results by directly evaluating the trajectories and discarding the unrealistic ones. In a test conducted by Tauro et al. [73] on a dataset of 12 videos captured in a natural stream, the accuracy of the PTV results was higher than that of LSPIV. Considering that different available algorithms are subject to errors, allowing for verification that the trajectory shapes and directions are realistic is of great importance for the accuracy of the final results.

### 3.2. Algorithms Based on Space-Time Images of Water Flow

**Figure 7:**
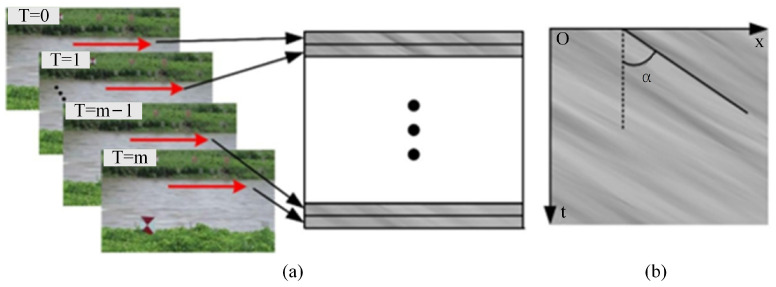
Generation of the STI. (**a**) The process of the STI generation. The red line with an arrow represents the velocity-measuring line set along the flow direction. The black and white panels represent the GCPs. T = 0 represents the first frame of the video and so on. (**b**) The generated STI of the velocity-measuring line indicated in (**a**) [4].

Fujita et al. [3] introduced the water surface spatio-temporal image into river measurements for the first time and detected the main direction of texture in the spatio-temporal image using the Gradient Tensor Method (GTM) to obtain the one-dimensional time-averaged surface velocity, which was named STIV. Compared with LSPIV, the significant progress of this method is that it does not need to use tracer particles. Instead, it relies on the formation of a space-time image using water surface flow features, such as ripples and corrugations. The surface flow velocity is determined by analyzing changes in grayscale on the image. The process of STIV is as follows: a series of parallel and equal-length velocimetry lines are set up along the direction of the water in the captured video of the river flow and the gray level of each velocimetry line is extracted frame-by-frame from the video. The grayscale information of each line is arranged sequentially to synthesize the space-time image of the line. In each space-time image, the change in the brightness of the river surface will be presented as an approximate parallel band texture. Then, the size of the surface velocity can be deduced by solving the angle between the band texture and the vertical direction (Texture Angle) [4], as shown in Figure 7.

The central issue in solving the flow velocity using STIV is the main texture angle of the image. Various methods have been proposed for solving the angle, such as GTM, the Two Dimensional Autocorrelation Function (QESTA) [26], and FFT-based Space-Time Image Velocimetry (FFT-STIV) [74]. The texture angle is computed in GTM based on the relation of the local grey scale integrals of the image. In QESTA the grey scale distribution of the image in polar coordinates is solved first, and the texture angle corresponds to the location of the maximum intensity of the function along the angular direction. In FFT-STIV, the main texture information is obtained by identifying the high-frequency part of the image frequency domain. These methods have been applied in the investigation of actual field river discharge measurements, but they have certain requirements for the observation environment. After identifying the texture angle, Equation (7) is used to calculate the surface flow velocity:

(7) $$v=\tan\alpha \cdot S_{x}\cdot fps$$

where *α* is the size of the texture angle (unit: degree), *S_x_* represents the resolution of the velocimetry line (unit: m/pixel), which is calculated during the camera calibration, and *fps* is the frame rate of the camera (unit: frames/s), which is generally a constant value and is related to the model of the camera used for shooting.

As a one-dimensional velocimetry method, STIV is not suitable for a turbulence-characterization study, flow pattern study, vorticity study, and other fields that require fine flow field portrayal. Yet, for flow tests that only need a one-dimensional velocity distribution, the STIV method demonstrates better performance: high spatial resolution to the single-pixel level and high computational efficiency that is more than 10 times that of LSPIV [75]. These unique advantages have led to its rapid development and application since it was proposed. The core of STIV is the correct identification of the texture angle, and a great deal of work has been carried out by various research teams in terms of improving the identification accuracy [4,76,77,78]. Considering the insufficient ability of STIV to estimate the two-dimensional flow field in rotating flows, Yu et al. [79] proposed a new angular identification algorithm, CASTI, and carried out a preliminary validation; Tsuji et al. [80] developed the Space-Time Volume Velocimetry (STVV) technique in which the recognition dimension of STIV was expanded; Legleiter et al. [81] attempted to generalize the STIV framework to make it feasible for two-dimensional reach-scale applications. Al-mamari et al. [82] and Kim et al. [83], on the other hand, evaluated the applicability of STIV in special discharge measurement scenarios of flash floods in arid zones and rotating wavebands. There are still many research teams exploring the method in depth.

### 3.3. Algorithms Based on Probability and Variational Methods

In the field of flow field determination, image processing methods based on optical theory occupy an important position. The dense optical flow method is employed to address the challenge of detecting moving objects, while the sparse optical flow method, originally proposed by Lucas and Kanade [84], substantially enhances the computational efficiency of the algorithm. Lin et al. [85] used hot water as a tracer, and the LK optical flow method can be utilized to achieve better small-scale discharge measurement results. In recent years, several algorithms for analyzing river velocity, by applying probabilistic and variational methods in combination with optical flow techniques, have also become rapidly popular. A comparison of the algorithms is shown in Table 2.

Probabilistic or variational-based algorithms are centered on the computation of the optical flow field, converting the problem of solving the velocity vector field into the problem of solving the extreme value of the objective function in fluid dynamics. These algorithms do not need tracer particles to solve the global motion vectors, but they are more dependent on a priori knowledge of the flow field and have higher computational costs. For the probability-based velocimetry methods, the difficulty lies in the selection of the a priori model and likelihood model; different likelihood models and the improved hyper-parameter estimation have been proposed [90]. The difference in the variational-based methods is mainly reflected by the design of the data terms and constraints, and how to optimize the selection needs further research.

### 3.4. Algorithms Based on Deep Learning Methods

**Figure 8:**
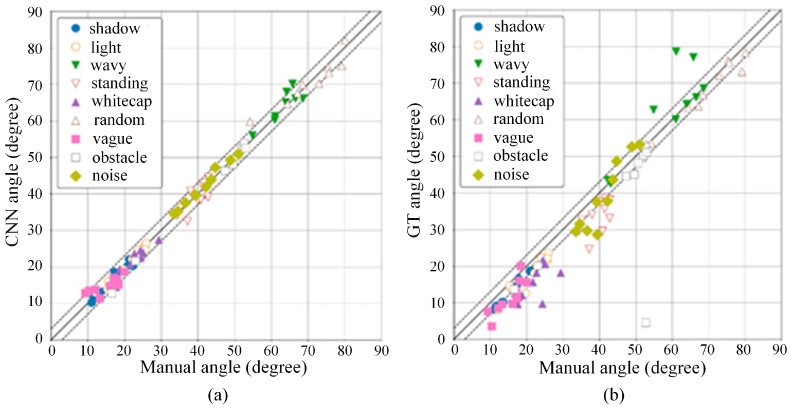
Comparison with manually confirmed angles: (**a**) CNN; (**b**) gradient tensor method [29].

Deep learning methods represented by convolutional neural networks (CNNs) show excellent ability in recognizing image geometric features and then constructing river surface velocity recognition models. The image recognition model constructed by CNN directly uses a computer instead of a human brain to perform machine learning on a large number of water flow picture datasets (with possible pseudo samples). The identification process can be divided into the following steps: (1) determine the velocity labels corresponding to the water flow pictures, (2) adjust the structural parameters through the back-propagation algorithm to accurately divide the feature space, (3) automatic extraction of texture features of the water flow surface under different velocity levels, (4) classification of the velocity, and (5) finally realize the real-time and efficient conversion between water flow pictures and velocity information. Ansari et al. [91] developed a river velocimetry scheme called RivQNet through FlowNet architecture, which can obtain accurate and dense spatial distributions of surface velocities without user inputs. Wang et al. [92] proposes a discharge measurement method based on the recurrent all-pairs field transforms for optical flow (RAFT) algorithm, which introduces a convolutional block attention module and deformable convolution to enhance the ability to capture complex flow surface information. Watanabe et al. [29] introduced a deep learning method into the STIV to classify texture angles, which improves the accuracy and robustness (Figure 8).

Deep-learning-based velocimetry methods for river surfaces are still in their infancy and exhibit several shortcomings. These include coarse classification and the recognition interval of flow velocity, low accuracy, and high requirements for parameter selection and the dataset size. Nevertheless, these methods excel in feature extraction, and their performance can be significantly enhanced with a larger and more diverse dataset, offering promising prospects for future applications in velocimetry.

The above four classes of algorithms essentially recognize the surface velocity by converting it into some observable feature. The velocity is calculated from displacement of the corresponding feature in a video frame interval. Algorithms based on particle image recognition use artificial tracers, while the other algorithms recognize features of the water flow itself, such as ripples. Algorithms based on space–time images of water flow only consider one-dimensional directional flow velocity, but with high accuracy, while algorithms based on particle images recognition, probability, and variational methods give a better estimation of the flow field motion. Algorithms based on deep learning methods have little experience, but perform well when used in combination with other algorithms and have advantages in data classification. There are a number of variants of all types of algorithms, and in general, the accuracy and adaptability of the algorithms are improving.

## 4. Challenges in Video-Based River Discharge Measurements

### 4.1. Image Acquisition Quality

The accuracy of the results in velocimetry is heavily reliant on the quality of image acquisition. However, this quality is inherently limited by the characteristics of the flow and the capabilities of the capture equipment.

Using video flow measurements for long-term velocity observations has the potential to enable real-time, automated flow monitoring, which could address a significant challenge for individual hydrological stations. However, like other flow-measurement techniques, image velocimetry algorithms have difficulty in accurately identifying very-low-flow velocities. This is because the velocities are too low to drive an artificial tracker forward or to form distinct ripples on their own to provide geometric features for image-recognition algorithms. This means that when the river is in its off-season there is a risk of invalid flow images. In addition, due to the limitation of the imaging range of the camera, it is difficult to obtain cross-section images of large rivers using a complete set of equipment.

For artificial tracers, their visibility in the complex water surface optical environment of a live river is poorer than in the laboratory. In addition, their uneven spatial and temporal distribution will cause time-averaged flow field reconstruction errors, affecting the recognition effect. Similarly, changes in the tracer particle size, surface coating, and other parameters can also interfere with the recognition of velocity. Hadad and Gurka [93] investigated the effect of tracer particle parameters in the PIV/PTV method under turbulent flow conditions. They measured the velocity field in a standard flow with different parameters and statistically examined the effect on the turbulence volume and the velocity derivative. It was found that at higher Reynolds numbers, the effect of particle parameters is greater; particle size has the greatest effect on velocity and acceleration, and the particle concentration and chemical treatment do not affect the results independently, but there is some effect when they are changed at the same time. It is necessary to find new artificial tracers for particle dispersal to maximize the flow characteristics without destroying the natural river environment.

On the other hand, the process of image analysis is dependent on the hardware platform. The selection of parameters, such as particle identification, the search area, and the rate of frame extraction is limited by the camera model, data volume, and storage device. High-definition cameras are convenient for detailed measurements of large-scale rivers but require a large amount of computer memory for storage [24].

### 4.2. Environmental Conditions Limitations

**Figure 9:**
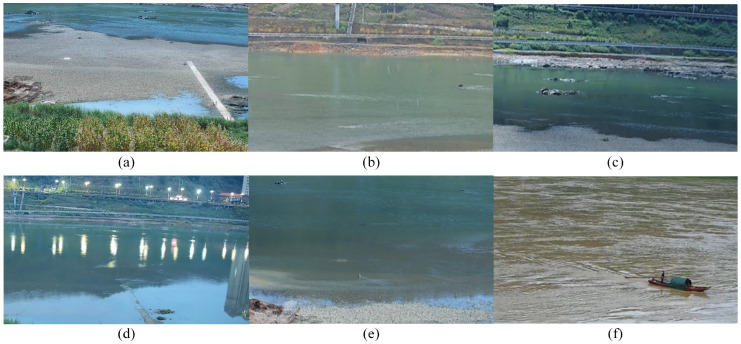
Some typical unfavorable environmental conditions: (**a**) strong wind; (**b**) rainstorm; (**c**) uneven field illumination; (**d**) night; (**e**) bird activities; and (**f**) human activities.

The unfavorable factors arising from complex meteorological conditions and watershed environments can greatly reduce measurement accuracy or prevent measurements altogether [4,79]. Some typical unfavorable conditions are shown in Figure 9.

For meteorological conditions, particularly wind fields (Figure 9a), can interfere with the measurements by affecting the water surface flow velocity vector. And strong winds can disrupt the water flow and even damage the filming equipment by swathing branches and rocks in the vicinity. Muste et al. [94] found that a constant wind on the free surface can be successfully used as a tracer for an “unseeded” flow over a wide range of dynamic conditions. Therefore, they considered that by modeling the wind field and making an analysis, wind-affected measurements can be compensated to provide reliable estimates of water body movements. In the case of heavy rainstorms (Figure 9b), raindrops and water spray can reduce video visibility, making it virtually impossible to obtain valid images. Different lighting conditions also have complex effects on measurement results. Uneven field illumination (Figure 9c) and its variations in daylight create background noise, resulting in weak contrast and dark spots on the image. Simple correction takes the form of global histogram equalization and local adaptive histogram equalization, and NIR imaging combined with eigen mean-difference fusion methods can also be effective in overcoming this effect. Detert [95] recommended the choice of cloudy weather conditions with diffuse light for uniform illumination. Darker light, e.g., cloudy days or nights (Figure 9d), will undoubtedly affect the video visibility to locate the position of the tracers or to identify the gray-scale representation of the ripples. Especially at night, the image is almost completely black and the video becomes a still image. The use of a far-infrared camera is an effective improvement, which mainly detects the temperature difference, as well as the radiation intensity that varies with the roughness shape, and can be operated both during the day and at night [96]. Fujita [19] compared advection ripple velocities identified at night in the same place with an ordinary camera and a far-infrared camera and found consistent results. Although the resolution of the far-infrared camera is low, it still provides good analysis if the image sharpness is increased, and a high-resolution camera will further improve accuracy [19,96]. Methods, such as Multi-Scale Retinex (MSR) and Fourier Maximum Angle Analysis (FMAA) [47] that help to enhance image contrast and filter out noise, can be used as a means to increase the image sharpness and clarity of image recognition, improving accuracy and robustness in uneven lighting and nighttime observations.

In general, for complex meteorological conditions with high disturbance intensity and short duration, such as strong winds, heavy rain, fog, snow, and ice, it may be wisest to avoid measurements during that period, when any measurement method is unsafe and inaccurate. For more stable adverse meteorological conditions, some strategies can be considered to improve the impact on the measurement accuracy. A constant wind field or uniform rainfall is expected to be reasonably well modeled, with the wind speed vector or raindrop interference disentangled from the measured results [94]. A simple correction for uneven field illumination is histogram equalization, and new methods, such as multi-scale dynamic fusion, are also worth trying [97]. In addition, supplemental lights can be considered for poor lighting conditions to provide sufficient light when needed but may be less useful in riparian environments with heavy vegetation and mosquitoes [98]. For night environments, Fujita [19] achieved nighttime observation based on the STIV by introducing a far-infrared camera, but the nighttime measurement problem has still not been solved after that. The thermal imaging technique also needs to overcome the challenges of cost, field of view, and durability [99]. Therefore, the development of a new generation of infrared cameras with higher resolution, a larger field of view, and lower cost will help to further improve the applicability of video-based methods.

For aquatic environments, the width of the river is always a prerequisite for the use of video discharge measurements. In the case of wide rivers, elevating the camera mounting height is an option to achieve a broader field of view. Nevertheless, challenges persist in accurately measuring flow velocities of the entire cross-section due to image resolution limitations. For this reason, Fujita et al. [100] used different pitch angles for shooting and employed GCPs and water surface control points to match and integrate the images acquired from different river widths. This innovation expanded the effective river width capture capacity of the STIV method from over 100 m to more than 300 m.

In the context of high-water conditions, surface flow velocity measurements are also susceptible to errors. These errors arise from both small sample sizes and adverse imaging conditions, as well as environmental disturbances.

Finally, biological activities are a category of infrequent, yet highly impactful, adverse factors. The organisms here mainly focus on the birds (Figure 9e), animals inhabiting and moving within the river basin, and human beings (Figure 9f). Birds and animals may access the measurement area for drinking, while humans may paddle through the measurement section or engage in fishing, thereby influencing the flow of tracers or compromising the integrity of the tracer patterns. The field measurement environment determines that it is difficult to completely avoid such situations. Therefore, it becomes imperative to diminish the probability of occurrence through the measures, such as the judicious selection of measurement points and the implementation of pertinent physical isolation measures.

### 4.3. Uncertainty of Parameters

In addition to the challenges mentioned above, video-based velocimetry methods also face another risk: the uncertainty of parameters. The process of obtaining a significant number of parameters is not completely rigorous, and their true values are actually unknown, and this uncertainty also leads to the inaccuracy of parameters. It is discussed here in two categories: inaccuracy of ranging model parameters and inaccuracy of algorithm parameters.

The inaccuracy of ranging model parameters arises from the errors generated by the flow field calibration, especially in the inclined view. Reference objects or GCPs need to be arranged in the field for velocity calibration before the flow measurement. Liu et al. [101] found that in the case of a single GCP measurement, the effect on the calibration parameters is significant, i.e., shifts and wider confidence intervals, and that this uncertainty can be mitigated if the coordinates of the control point are measured in three repetitions and averaged. Schweitzer and Cowen [102] analyzed the spatial uncertainty in image velocimetry and found that the uncertainty in velocity measurements associated with direct georeferencing was five times larger than when using GCP-based georeferencing in an oblique camera view. For a more in-depth study of ranging models, Le Coz et al. [103] developed a Bayesian approach that allows for a combination of a priori information about camera parameters with observations of real-world and image coordinates of a limited number of GCPs to improve the measurement accuracy. Zhang et al. [104] introduced direct sensor oriented (DSO) photogrammetry into the STIV method and designed a photogrammetric device supporting a laser distance meter (LDM) to realize a GCP-free surface velocity measurement under an oblique shooting angle. This team proposed that the water level change under an inclined viewpoint may have the following effects on the calibration accuracy: (1) the measured water surface height is dynamically changing, the amplitude of water level changes of mountain streams and rivers during high floods can be up to several meters in a short time; (2) it is difficult to lay coplanar control points on the measured water surface; (3) the camera is usually tilted at an angle of view to shoot a large view field of the water, and the perspective aberrations will lead to the uneven distribution of spatial resolution; (4) the aberration of the optical system of a non-measuring camera cannot be ignored; especially, when a wide-angle lens is used, the aberration of pixels far from the center of the image is more pronounced.

**Figure 10:**
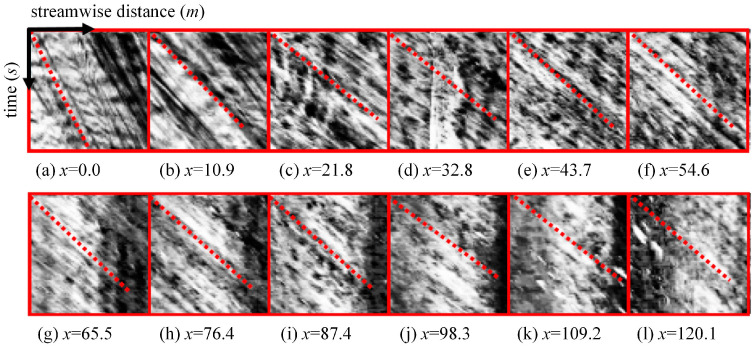
Space–time images obtained with a high-definition camera at several transverse locations (unit in meter): horizontal scale is 10.9 m and downward vertical scale is ten seconds. Images are enhanced for a better recognition. The dotted line is the main orientation angle of the pattern. [19].

The inaccuracy of algorithm parameters is reflected by the subjective assignment of certain parameters by the researchers, often reliant on experiential judgments, resulting in coarse parameter values. Further in-depth research in this area is warranted. For STIV, the window size parameter in the GTM and the intensity coefficient parameter M in the QESTA are both generally taken as 15. However, the same space–time image can be divided into different numbers of windows with different window sizes. The region where each window is located is not the same each time, and the textures contained in them are not the same. An STI section with a window width of 10.9 m is shown in Figure 10, where a difference in the main texture orientation of each small window can be seen. Thus, the recognition of the angle obtained is inevitably not the same when taking different values of the window size. Similarly, taking different values of M results in different strengths of the logarithmic polar transformation relationship, resulting in a change in the gray value of the image. This indicates that the parameter values will have a great impact on the angle recognition results, and the optimal values of the parameters are not fixed; different image features will have different optimal parameters. Therefore, the velocity recognized by the GTM and the QESTA may not be truly accurate. For the conversion of velocity to discharge, the surface velocity coefficient α is also empirically taken and cannot match the individualized needs of each monitored river [82]. Therefore, the magnitude of the possible error cannot be predicted. For deep learning algorithms, the accuracy of their parameters is related to many factors, such as the size of the dataset, the representativeness of the dataset, and the degree of model training. In addition, considering the complex flow characteristics of rivers and the variable outdoor measurement environment, outliers in the calculation results are almost inevitable. How to deal with the calculated outliers according to different measurement situations is still a problem worth studying and discussing.

In general, the following strategies can be considered to optimize parameters in practical measurements to enhance measurement accuracy and computational efficiency: (1) for the optimization of ranging model parameters, a new 3D reconstruction algorithm can be used. Combined with a priori information of the camera, the overall calibration is carried out and passes the repetition test. Then, the correction term is introduced to construct a nonlinear mapping relationship from image coordinates to physical coordinates to overcome the dynamic changes in water surface elevation and the influence of optical distortion of the camera on the results [101,103,104]; (2) for the optimization of algorithm parameters, firstly, the shape of the measurement section and the river flow pattern need to be considered to select the appropriate image-recognition algorithm, and secondly, the algorithm itself should be continuously improved by replacing or removing the original parameter that is roughly valued or by adopting a more rigorous and refined parameter valuing principle. For example, the error caused by the window size parameter in STIV can be removed by replacing the original processing method with the Hanning window function, which does not contain any calculation parameters. The important basis for refinement of the parameter value principle is the environmental factors, represented by the surface velocity coefficient used in the discharge calculation. Hauet et al. [105] concluded the following after analyzing 3611 gauging stations on 176 rivers: (1) the surface velocity coefficient is positively correlated with the water depth, while the relationship with the roughness is very complicated, and the specific relationship is still unclear; (2) for natural rivers, the average value of the coefficient is 0.8; (3) for artificial concrete channels, the mean value of the coefficient is 0.9. In addition, the surface velocity coefficient varies with the channel aspect ratio, vegetative conditions, tidal water level fluctuations, and the presence of secondary flows [32,106,107]. The complexity of the factors influencing the surface velocity coefficient means that the true value for a river is virtually unknown, and current studies have typically used 0.85, which is close to the true value for most rivers [108,109,110]. A comprehensive value principle that can combine multiple influencing factors is waiting to be further summarized.

## 5. Conclusions

As a novel instrument for non-contact water body flow testing, the video-based river discharge measurement methods offer advantages, such as rapid measurements, cost-effectiveness, and comprehensive monitoring capabilities. The safe and effective observations of river surface velocity and discharge are possible by using the video-based instrument during a flood period, thereby demonstrating substantial potential for broad development. Since LSPIV was proposed, scholars have developed image-recognition velocimetry methods based on particles, space–time images of water flow, and probabilistic, variational, and deep learning. River discharge is then obtained using the velocity-area method. At the same time, some factors constrain the full potential of these techniques, such as deficiencies in the algorithms themselves, poor image-acquisition quality, uncertainty of parameters, complex meteorological conditions, and watershed environments.

It is suggested that future research can follow four research ideas as follows; (1) further develop image recognition algorithms with higher accuracy, robustness, and computational efficiency, advance the development and deepening of deep learning in the research of video-based discharge measurement techniques; (2) establish a mapping model between extreme weather conditions, such as the wind field, rainstorms, and the results of image recognition, quantitatively calculate the size of the impact of wind and rain on the surface velocity field, and further explore the observation methods at night; (3) deepen the mechanistic study of river discharge measurements and further clarify the value principle of the surface velocity coefficient in different rivers, different water levels, and different hydrological characteristics; and (4) synthesis of theoretical research results to develop a mature, usable, and cost-controllable integrated video flow measurement system with visualization capability and user-friendly attributes and realize large-scale application in discharge testing work.

## Figures and Tables

**Table 1:** Summary of research to improve LSPIV.

Direction	Research	Contents
Enhancing the accuracy of flow velocity measurements	Scarano and Riethmuller [63]	The Window Deformation Iterative Multigrid (WIDIM) was proposed, improving the correlation analysis method.
	Weitbrecht et al. [64]	WIDIM was introduced to LSPIV, using the velocity computed in a large query region as a reference for the next smaller region and iteratively computed.
Improving the real-time and robustness of measurements	Zhang et al. [65]	An LSPIV system based on an IP camera was developed, and a nonlinear processing strategy was used to reconstruct the time-averaged flow field.
Improving computational efficiency	Dobson et al. [62]	The Fast Fourier Transform Cross Correlation (FFT-CC), a frequency-domain method, was used to implement the LSPIV computation to maintain a high processing performance.
Sensitivity analysis and uncertainty assessment	Meselhe et al. [66]	The sensitivity of tracer particle density in a laboratory sink was analyzed.
	Hauet et al. [67]	The sensitivity of the environmental parameters, the internal and external parameters of the camera, and the parameters of the analysis algorithms were systematically evaluated.
	Le Coz et al. [55], Harpold et al. [68]	The factors influencing the accuracy of LSPIV were obtained through a flow comparison measurement test.
	Muste et al. [24]	27 elemental sources of error were pooled, concluding that their relative contributions to the final results could be ranked in the following order: seeding density, identification of the GCPs, accuracy of flow tracing by the seeding particles, and sampling time.
Proposing new LSPIV-based velocimetry approaches	Leitao et al. [69]: Surface Structure Image Velocimetry (SSIV)	Apply localized filtering for the post-processing of the flow field, and removing vectors with large deviations from neighboring velocity vectors, as well as from the globally averaged vector, to correct the results.
	Admiraal et al. [70]; Baek and Lee [71,72]: LSPTV	Rely on different particle-tracking algorithms (related to the number of image frames used) to recognize and track individual particles and reconstruct particle trajectories; suitable for images with low particle concentration.

**Table 2:** The comparison of discharge measurement algorithms based on probability and variational.

Research	Algorithm	Year	Overview	Advantages	Disadvantages
Bacharidis et al. [86]	Probability-based method	2014	Build a system framework for probability-based image discharge measurements. Given the image observation function, calculate the posterior distribution of each pixel using a Bayesian framework to maximize the velocity vector.	Higher accuracy when dealing with non-smooth modes, dense optical flow field	Need to improve the accuracy of optical flow and classification algorithms, optimize the likelihood estimates describing conditional probability distributions
Perks et al. [87]	KLT-IV	2016	Use spatial intensity information to detect and search for locations that produce the best match. Each Tomasi corner point translation or affine is tracked between consecutive frames and connected to obtain a motion trajectory.	Works well under conditions of sparse surface features and in unsteady flow	Sensitive to changing the feature-extraction rate
Tauro et al. [50]	OTV	2018	Surface flow observations using optical flow and trajectory-based filtering. Corner features are automatically detected with the Fast algorithm, features are tracked using the sparse variational LK algorithm, and posterior-based trajectory filtering is performed.	Less affected by noise and surface seeding, computationally efficient and robust	Highly affected by unstable frame acquisition frequency and low-velocity field resolution
Khalid et al. [88]	SGSD	2019	Physical constraints are added to the variational optical flow framework. The optical flow field is calculated using the scalar transport equation, and a weighted diffusion term is introduced to supplement the small-scale flow field features. On the basis of it, reconstruct the characteristic particle trajectories.	The velocity vector field is calculated with high accuracy	High river flow velocity is required, and the reconstruction of flow trajectories is susceptible to outliers
Lu et al. [89]	FS-VOF	2019	A variational spectral flow method based on field segmentation. The particle image is segmented according to the velocity distribution of the fluid flow, and multiscale image warping operations are performed in each subregion.	The spatial discontinuity property of the non-uniform flow field can be maintained, and a more accurate velocity field can be obtained	For non-uniform flow fields; it is better to include a segmentation term in the method
Huang et al. [1]	L^1^-Diffusion method	2022	A method for estimating the image plane flow field by attaching a regularization term to the convective diffusion equation. Choose L1 paradigm as the basis of the energy function. The energy-generalized minimum is solved using the LK method and other methods.	The computational results are highly accurate and robust, adapting to different hydraulic conditions and image shooting angles	More research on the selection of data terms and regularization terms is pending
